# Representation Learning: Recommendation With Knowledge Graph *via* Triple-Autoencoder

**DOI:** 10.3389/fgene.2022.891265

**Published:** 2022-06-03

**Authors:** Yishuai Geng, Xiao Xiao, Xiaobing Sun, Yi Zhu

**Affiliations:** ^1^ School of Information Engineering, Yangzhou University, Yangzhou, China; ^2^ Department of Ultrasound, Affiliated Hospital of Yangzhou University, Yangzhou, China

**Keywords:** personalized recommendation, autoencoder, semi-autoencoder, representation learning, collaborative filtering

## Abstract

The last decades have witnessed a vast amount of interest and research in feature representation learning from multiple disciplines, such as biology and bioinformatics. Among all the real-world application scenarios, feature extraction from knowledge graph (KG) for personalized recommendation has achieved substantial performance for addressing the problem of information overload. However, the rating matrix of recommendations is usually sparse, which may result in significant performance degradation. The crucial problem is how to extract and extend features from additional side information. To address these issues, we propose a novel feature representation learning method for the recommendation in this paper that extends item features with knowledge graph via triple-autoencoder. More specifically, the comment information between users and items is first encoded as sentiment classification. These features are then applied as the input to the autoencoder for generating the auxiliary information of items. Second, the item-based rating, the side information, and the generated comment representations are incorporated into the semi-autoencoder for reconstructed output. The low-dimensional representations of this extended information are learned with the semi-autoencoder. Finally, the reconstructed output generated by the semi-autoencoder is input into a third autoencoder. A serial connection between the semi-autoencoder and the autoencoder is designed here to learn more abstract and higher-level feature representations for personalized recommendation. Extensive experiments conducted on several real-world datasets validate the effectiveness of the proposed method compared to several state-of-the-art models.

## 1 Introduction

The success of machine learning algorithms and artificial intelligence methods heavily depends on the feature representation learning of original data (Bengio et al., 2013; Zhuang et al., 2017a). In recent decades, feature representation learning has attracted a vast amount of attention and research from multiple disciplines, such as biomedicine and bioinformatics (Wei et al., 2019; Li et al., 2021), computer vision (Kim et al., 2017), knowledge engineering (Liu et al., 2016), and personalized recommendation (Zhuang et al., 2017b; Zhu et al., 2021). In real-world applications, feature representation learning is considered to obtain the different explanatory factors of variation behind the data (Locatello et al., 2019).

For nearly three decades, effective computational methods have accelerated drug discovery and played an important role in biomedicine, such as predicting molecular properties and identifying interactions between drugs/compounds and their target proteins. In early years, quantum mechanics (Hohenberg and Kohn, 1964), such as density functional theory (DFT), was used to determine the molecular structure and calculate properties of interest for a molecule. However, the quantum computational method usually consumes tremendous computational resources and takes hours to days to calculate the molecular properties (Ramakrishnan et al., 2015), which hinders their applications to the fields of high-throughput screening. Nowadays, the powerful ability to learn representation and efficiently recommend algorithms has received significant attention. A key challenge is to learn useful molecular representation information from the huge molecular dataset.

Among all the informatics-related application scenarios, with the rapid development of the Internet, there is an urgent demand for personalized recommendation to tackle the information overload problem (Zhang et al., 2017). Notably, many successful recommendations systems share aspects of feature representation learning and have been widely applied in many online services such as electronic commerce (Ma et al., 2020) and social networks (Botangen et al., 2020). Existing methods for recommendation systems can roughly be categorized into three classes: content-based recommendation, collaborative filtering (CF), and hybrid methods (Batmaz et al., 2019). The content-based recommendation methods learn the descriptive features of items, calculate the similarity between new items and user-liked items based on these features, and generate the final recommendation (Lops et al., 2019). The collaborative filtering methods discover the inclinations of users by considering the user’s historical behavior and produce recommendations (Dong et al., 2021). Hybrid recommendation methods leverage multiple approaches together and try to combine the advantages of these approaches.

Recently, collaborative filtering methods have achieved superior performance for the advantages of effectiveness and efficiency, which have far-ranging consequences in practical applications of recommendation systems (Su and Khoshgoftaar, 2009). Most of the traditional collaborative filtering methods are based on matrix factorization (MF), which combines good scalability with predictive accuracy (Luo et al., 2020). The main intuition behind these approaches is to decompose the rating matrix into user and item-based profiles, which allows the recommendation system to treat different temporal aspects separately (Yehuda et al., 2009). However, MF-based methods have inherent limitations in feature representation learning for the recommendation, which prevent further development of these approaches.

On the other hand, deep learning techniques have recently achieved great success in the computer vision and natural language processing fields. Such techniques show great potential in learning feature representations. Therefore, researchers have begun to apply deep learning methods to the field of recommendations (Salakhutdinov et al., 2007). They use a restricted Boltzmann machine instead of the traditional matrix factorization to perform the CF, and Georgiev and Nakov,(2013) expanded the work by incorporating the correlation between users and between items. In addition, Wang et al. (2015), proposed a hierarchical Bayesian model that uses a deep learning model to obtain content features and a traditional CF model to address the rating information. These methods, based on deep learning techniques, more or less make recommendations by learning the content features of items. These methods are not applicable when we are unable to obtain the contents of items. Therefore, enhancing the effectiveness of feature learning is significant. Recent studies have shown that deep neural networks can learn more abstract and higher-level feature representations (Yi et al., 2018), which has made remarkable progress in improving recommendation performance (Chae et al., 2019). For example, He et al. (2017) proposed a general recommendation framework called Neural Network-based Collaborative Filtering, in which a deep neural network is utilized for learning the interaction between user and item features. As we can see, among all the deep neural network-based recommendation methods, many frameworks are realized on top of the autoencoder model, which is one of the most successful deep neural networks and has also been actively adopted as a CF model recently (Shuai et al., 2017; Zhuang et al., 2017c; Chae et al., 2019; Zhong et al., 2020). For example, Zhang et al. proposed a hybrid collaborative filtering framework based on an autoencoder that incorporated auxiliary information for semantic rich representations teaching (Shuai et al., 2017).

Though the autoencoder-based methods have achieved fairly good performance for personalized recommendation, there are two main problems that prevent the further development of these methods. The first is the utilization of auxiliary information from users or items, since the rating matrix in real-world applications is usually very sparse, which inevitably leads to a significant recommendation performance degradation. Most existing methods only introduce some obvious attributes, such as the age, gender, and occupation of users, or the title, release date, and genres of items. The key factors of collaborative filtering, such as the reviews of items by users, have rarely been incorporated into the autoencoder-based networks. The second problem is the optimization of neural networks. When training models to incorporate side information about items and users, the dimensions of the input and output layers are required to be equal in autoencoder-based networks, which greatly limits the scalability and flexibility of networks.

To address these problems, we propose a feature representation learning method for personalized recommendation in this paper which extends items features with knowledge graph via triple-autoencoder (KGTA for short). Specifically, the comment information between users and items is first encoded as sentiment classification. These features are then applied as the input to the autoencoder for generating the auxiliary information of items, which can be used to introduce the comment information from users to items to solve the incorporating problem of auxiliary information. Secondly, the item-based rating, the side information, and the generated comment representations are incorporated into the semi-autoencoder for reconstructed output. It aims to address the second problem, that the dimensions of the input and output layer are required to be equal. Finally, the reconstructed output generated by the semi-autoencoder is input into a third autoencoder for personalized recommendation. Experimental results on several datasets demonstrate the effectiveness of our proposed method compared to other state-of-the-art matrix factorization methods and deep-based methods.

In summary, the main contributions of our work can be distilled into the following:

• To incorporate the key information between users and items, the comments from each user for item are encoded and reconstructed as the auxiliary information
• To optimize the neural networks, a serial connection of semi-autoencoders and autoencoders are designed to learn more abstract and higher-level feature representations for personalized recommendation
• Extensive experiments on several datasets were conducted to confirm the effectiveness of the proposed method compared to other state-of-the-art matrix factorization methods and deep-based methods

## 2 Related Work

In this section, we survey the related works of feature representation learning, personalized recommendation methods, and collaborative filtering^1^ ^,^ ^2^.

### 2.1 Feature Representation Learning

Feature representation learning refers to learning data representations that make it easier to extract useful information in downstream machine learning tasks (Bengio et al., 2013). The last decades have witnessed a vast amount of research and application on feature representation learning in multiple disciplines. For example, in the field of biomedicine and bioinformatics, Wei et al. (2019) developed a bioinformatics tool for the generic prediction of therapeutic peptides. An adaptive feature representation learning method is proposed for different peptide types in the tool. Alshahrani et al. (2017) proposed a knowledge representation learning method with symbolic logic and automated reasoning, which can be applied to biological knowledge graphs for tasks such as finding candidate genes for diseases and protein-protein interactions. Li et al. (2021) proposed a triplet message mechanism to learn molecular representation based on graph neural networks, which can complete molecular property prediction and compound-protein interaction identification with few parameters and high accuracy.

Besides the fields of biomedicine and bioinformatics, feature representation learning has also been widely applied in other fields such as computer vision (Kim et al., 2017), knowledge engineering (Liu et al., 2016) and personalized recommendation (Zhuang et al., 2017b). For example, Wang et al. proposed a high-resolution representation learning network for visual recognition problems (Wang et al., 2020), which can maintain the representation being semantically strong and spatially precise. Xu et al. (2018) proposed an aggregation method for node representation learning that can adapt neighborhood ranges to nodes. It is especially suitable for graphs that have subgraphs with diverse local structures. Niu et al. (2020) proposed a rule and path-based joint embedding method for representation learning on knowledge graphs. The Horn rules and paths are leveraged in this method to enhance the accuracy and explainability of representation learning.

### 2.2 Personalized Recommendation

In recent decades, with the rapid development of the Internet, personalized recommendations have provoked a vast amount of attention and research (Qian et al., 2013). The advances in personalized recommendation have far-ranging consequences in many online services applications such as electronic commerce (Ma et al., 2020) and social networks (Li et al., 2017). For example, in Facebook, Gupta et al. (2020) conducted a detailed performance analysis of recommendation models on server-scale systems present in the data center. Botangen et al. (2020) proposed a probabilistic matrix factorization-based recommendation method that considers geographic location information for designing an effective and efficient Web service recommendation.

Good feature representations of data do contribute to many machine learning tasks, such as personalized recommendation. For example, Geng et al. (2015) proposed a deep method to learn the unified feature representations for both users and images. This representation from large, sparse, and diverse social networks obviously improves the recommendation performance. Liu et al. (2019) proposed a joint representation learning method for multimodal transportation recommendations, which aims to recommend a travel plan that considers various transportation modes. Ni et al. proposed a recommendation model based on deep representation teaching (Ni et al., 2021). It contained information preprocessing and feature representation modules to generate the primitive feature vectors and the semantic feature vectors of users and items, respectively.

### 2.3 Collaborative Filtering

In personalized recommendations, the collaborative filtering (CF) methods aim to discover users’ preferences through the interactions between users and items. Existing CF methods can be roughly categorized into two classes: matrix factorization methods and deep neural network methods.

In the matrix factorization methods, these methods have difficulty in processing sparse data and have poor generalization ability, but they have low time and space complexity and good scalability. Lee et al. proposed the classical non-negative matrix factorization (NMF) model (Lee and Seung, 2001), which can decompose the rating matrix into user and item profiles. Along this line, Sun et al. proposed a Probabilistic Matrix Factorization (PMF) model that scales linearly with the number of observations and performs well on very sparse and imbalanced datasets (Salakhutdinov and Mnih, 2007). In light of PMF, Salakhutdinov et al. also proposed a Bayesian Probabilistic Matrix Factorization (BPMF) model (Salakhutdinov and Mnih, 2008), which controlled model capacity automatically by placing hyper-priors over the hyper-parameters to avoid over-fitting. Koren proposed combining the factor and neighborhood models for a more accurate recommendation performance (Koren, 2008), which further extends the model to exploit both explicit and implicit feedback by the users. In recent years, to address the problem that the attributes of users are often scarce for reasons of privacy, Rashed et al. (2019) proposed a nonlinear co-embedding GraphRec model, which treats the user-item relation as a bipartite graph and constructs generic user and item attributes via the Laplacian of the user-item co-occurrence graph.

Recently, due to the powerful ability of deep learning methods, remarkable progress has been made in learning higher-level and abstract representations for personalized recommendations (Wang et al., 2015; Yu et al., 2019). These methods have nonlinear transformation and powerful representation learning ability, but poor interpretability, large data requirements, and extensive hyper-parameter tuning. For example, He et al. (2017) proposed a general recommendation framework that designs a deep neural network to learn the interaction between a user and item features. Meanwhile, to address the cold start problem and improve performance for personalized recommendations, Ni et al. (2022) proposed a two-stage embedding model to improve recommendation performance with auxiliary information. In this method, two sequential stages, graph convolutional embedding and multimodal joint fuzzy embedding, are designed to fully exploit item multimodal auxiliary information. Among all the deep learning methods for personalized recommendation, we realize many successful frameworks on top of the autoencoder, which is one of the most successful deep neural networks and has also been actively adopted as a CF model recently (Shuai et al., 2017; Zhuang et al., 2017c; Chae et al., 2019; Zhong et al., 2020). For example, Zhuang et al. (2017c) proposed a dual-autoencoder model for recommendation, which simultaneously learns the user-based and item-based features with the autoencoder model. Zhu et al. (2021) proposed a collaborative autoencoder model for personalized recommendation, which learns the hidden features of users and items with two different autoencoders for capturing different characteristics of the data.

## 3 Preliminaries

### 3.1 Autoencoder

The autoencoder model aims to minimize the distance between the input and the reconstructed output. The basic autoencoder network (Bengio, 2009) generally consists of an input layer, an output layer, and one or more hidden layers. Given the input as $x\in R^{m\times n}$ , when there is only one hidden layer, the encoding and decoding layer of autoencoder can be represented as follows:

$$\xi =f\left(Wx+b\right),$$ (1)

$$x^{\prime }=g\left(W^{\prime }\xi +b^{\prime }\right),$$ (2)

where $W\in R^{k\times m}$ , $W^{\prime }\in R^{m\times k}$ and $b\in R^{k\times 1}$ , $b^{\prime }\in R^{m\times 1}$ are the weighting matrices and bias vectors, respectively. *f* and *g* are the nonlinear activation functions of the encode and decode layers, respectively. In our experiments, the sigmoid and identity functions are introduced as *f* and *g*. The objective function of the autoencoder can be shown as follows:

$$\underset{W,b,W^{\prime },b^{\prime }}{\min}J_{r}={\left\|x^{\prime }-x\right\|}^{2}.$$ (3)

### 3.2 Semi-Autoencoder

In recent years, many autoencoder-based recommendation methods have achieved fairly good results with the advantages of no labeling requirement and fast convergence speed. However, the classic autoencoder model has the restriction that the dimensions of the input and the output layer must be equal, which has a great impact on introducing auxiliary information for solving the sparse problem of the rating matrix.

**FIGURE 1:**
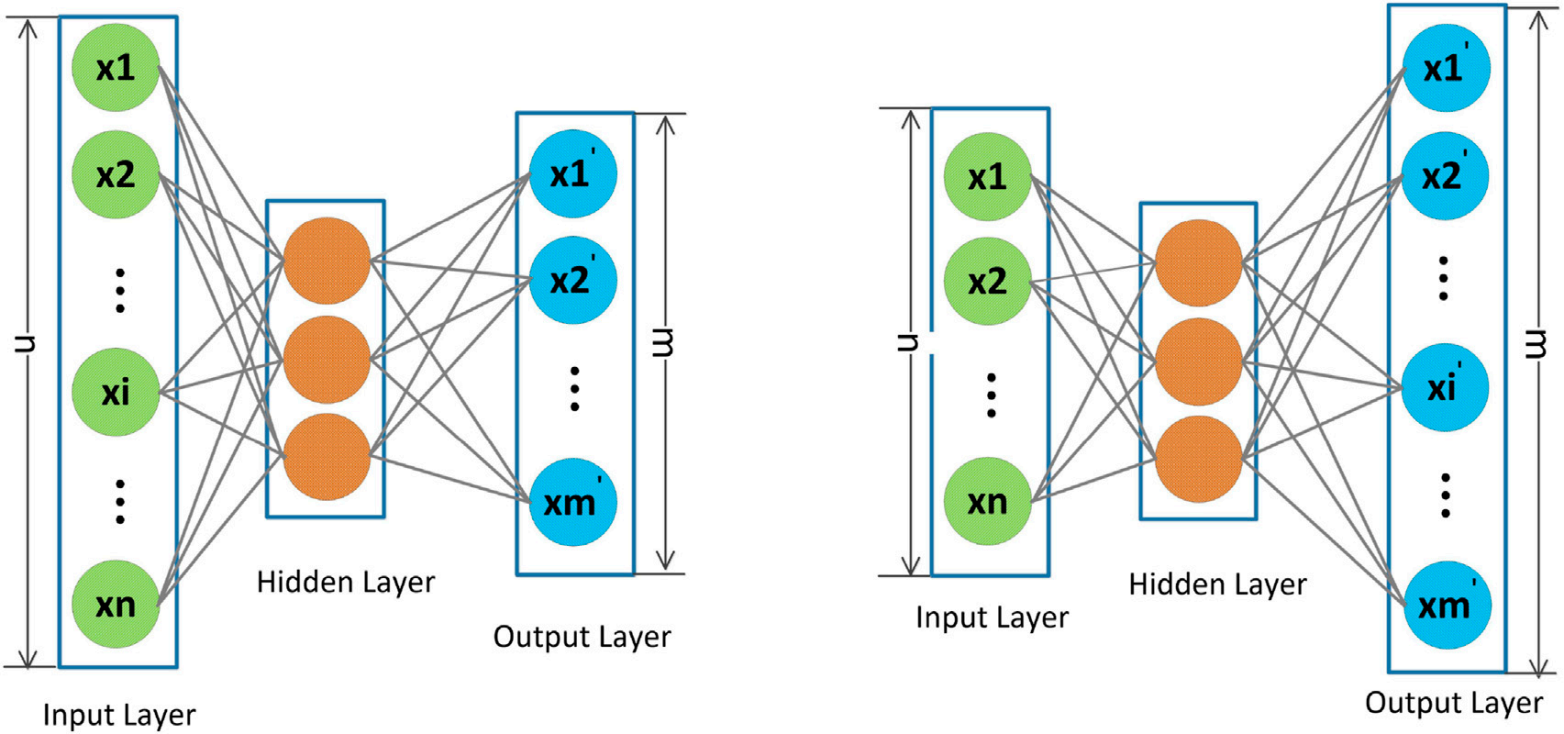
Illustration of a semi-autoencoder where the input and output layers can be inconsistent. The length of the input layer is longer/shorter than the output layer in the left/right part.

To address this problem, a semi-autoencoder model was proposed and generalized into a hybrid CF method for rating prediction (Shuai et al., 2017). Compared with traditional autoencoders, the input layer of semi-autoencoders is longer than the output layer, so semi-autoencoders can be utilized to capture different nonlinear feature representations and reconstructions flexibly by extracting different subsets from the inputs, and it is easy to incorporate side information into the input layer effectively to improve the item feature representation for better recommendation performance. The whole framework of the semi-autoencoder is shown in Figure 1, the left and right parts of Figure 1 show the two cases in which the output layer is longer than the input layer and the output layer is shorter than the input layer, respectively. We observe that the basic framework of a semi-autoencoder is the same as that of a classical autoencoder model, which also includes an input layer, an output layer, and one or more hidden layers. Furthermore, in the right part of Figure 1, we can observe that the shorter output layer is the reconstruction of certain parts of the input, and the remaining part in the semi-autoencoder model is auxiliary information to learn better feature representations for addressing the sparse problem of the rating matrix.

## 4 Methodology

**FIGURE 2:**
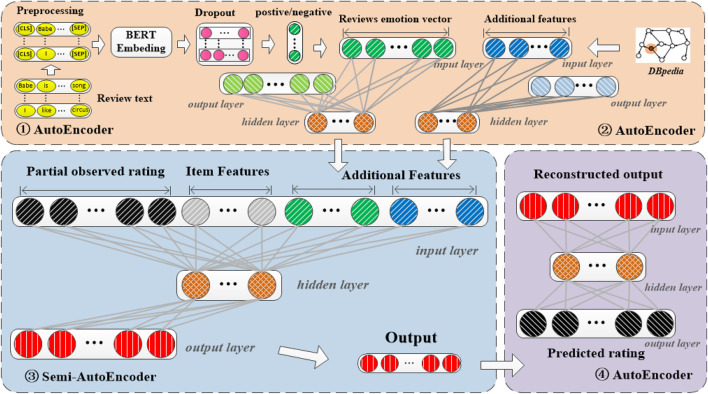
Whole framework of the proposed KGTA

The whole framework of our proposed recommendation method with knowledge graph via triple-autoencoder (KGTA for short) is illustrated in Figure 2, which encompasses three main components. The first one is the representational learning of the comment information between users and items. The comments from users on each item are divided into positive and negative categories. Then the first autoencoder was introduced to reduce the dimensionality of this comment information. The second one is the learning of all the auxiliary information. A semi-autoencoder is utilized to incorporate the side information, the extended features from the knowledge graph, and the generated comment features into the item-based rating. Finally, the low-dimensional output of the semi-autoencoder is input into the third autoencoder. Different from the semi-autoencoder model that only approximates the item-based rating; the third component tries to reconstruct all the input for the recommendation^3^ ^,^ ^4^.

In the following, first, the commonly used notations in this paper are listed in Table 1, and then, the model of KGTA is described in detail.

**TABLE 1:** Important notations used in this article and their descriptions.

Notations	Descriptions
*R*	The rating matrix
*A*	The attributes vectors of all items
*S*	The reconstructed comment vectors of all items
*L*	The language vectors of all items
*R*′	The prediction matrix *R*′ ∈ *R* ^*n*×*m*^
*m*	The number of users
*n*	The number of items
*r* ^*u*^	The column of rating matrix
*r* ^*i*^	The row of rating matrix
*k*	The features dimension of hidden units
*h*	The number of hidden units
*x*_*i*_	The *i*th instance of original input
$x_{i}^{\prime }$	The reconstructed output of *x*_*i*_
*ξ*	The hidden feature representation matrix
*W*, *W*′	The map and remap weight matrix
*b*, *b*′	The map and remap bias vectors
•	The element-wise product of vectors or matrices

### 4.1 Notations

Some important notations used in this paper and their descriptions are listed in Table 1.

### 4.2 Comment Information Features

The personalized recommendation is to predict the interest of a user in an item based on the rating matrix information. Since the rating matrix in real-world scenarios is usually very sparse, many methods have introduced auxiliary information to address this problem. However, most existing methods only introduce some obvious attributes and ignore the key factors, such as the comments from users on each item, of collaborative filtering. To address this problem, our method learns the comment information features between users and items with the first autoencoder. The details can be seen in the upper left of Figure 2.

In our method, we take natural language text as the input for sentiment classification and output emotion score $\in \left\{1,-1\right\}$ . −1 represents negative emotion and 1 represents positive emotion. Our method has two stages from input sentence to output score, which are described below.

In the first stage, we perform the following preprocessing steps on the comment text before we feed it into the model. First, we remove all the digits, punctuation symbols, and accent marks, and convert everything to lowercase. Secondly, we then tokenize the text using the WordPiece tokenizer (Schuster and Nakajima, 2012). It breaks the words down into their prefix, root, and suffix to better handle unseen words. Finally, we add the [CLS] and [SEP] tokens at the appropriate positions.

In the second stage, we build a simple architecture with just a dropout regularization (Srivastava et al., 2014) and a softmax classifier layer on top of the pretrained BERT layer. The upper left corner of Figure 2 shows the overall architecture of our sentiment classification model. There are four main stages. The first is the processing step, as described earlier. Then we compute the sequence embedding from BERT. We then apply a dropout with a probability factor of 0.1 to regularize and prevent over-fitting. Finally, the softmax classification layer will output the probabilities of the input text belonging to each of the class labels such that the sum of the probabilities is 1. The softmax layer is just a fully connected neural network layer with the softmax activation function. The output node with the highest probability is then chosen as the predicted label for the input.

Given the rating matrix $R\in R^{m\times n}$ , where *m* and *n* denote the number of users and items respectively. For each item, the comments from each user are classified by sentiment using BERT (Devlin et al., 2018) first, and then we obtain the comment feature vector *c*_*i*_ for each item. Since the comment information from users to items is usually sparse, just like the rating matrix, the first autoencoder was introduced for feature dimensionality reduction and representation learning. The process of the first autoencoder can be shown as follows:

$$\xi_{s}=f\left(W_{s}C+b_{s}\right),$$ (4)

$$s=g\left(W_{s}^{\prime }\xi_{s}+b_{s}^{\prime }\right),$$ (5)

where $W_{s}\in R^{k_{1}\times n}$ and $W_{s}^{\prime }\in R^{n\times k_{1}}$ are the weighting matrices, $b_{s}\in R^{k_{1}\times 1}$ and $b_{s}^{\prime }\in R^{n\times 1}$ are the bias vectors, *f* and *g* are the functions of nonlinear activation, and *k*_1_ is the feature dimension of hidden units. The hidden features of the first autoencoder, i.e., the low-dimensional representations of *s*, are denoted as *S* ^*I*^, which are incorporated into the second semi-autoencoder for capturing different representations and reconstructions by sampling different subsets from all the inputs.

### 4.3 Co-Embeddings With the Semi-Autoencoder

After obtaining the reconstructed comment features, a semi-autoencoder is introduced to incorporate the item rating vector *r*_*i*_ and other auxiliary information such as attributes vector *a*_*i*_, reconstructed comment features *s*_*i,*_ and the KG-extended features *l*_*i*_. The input of the semi-autoencoder can be defined as $con\left(r_{i},a_{i},s_{i},l_{i}\right)$

$$con\left(r_{i},a_{i},s_{i},l_{i}\right)=connection\;of\;r_{i},\;a_{i},\;s_{i},\;and\;l_{i}.$$ (6)

The $con\left(R^{I},A^{I},S^{I},L^{I}\right)\in R^{n\times \left(m+y+k_{1}+k_{2}\right)}$ refers to the connection of *R* ^*I*^, *A* ^*I*^, *S* ^*I*^ and *L* ^*I*^, where $R^{I}\in R^{n\times m}$ represents the item-based rating vectors, $A^{I}\in R^{n\times y}$ represents the attribute vectors of all items, which are the obvious attributes such as the title, release date, and genres in movie recommendation datasets, $S^{I}\in R^{n\times k_{1}}$ represents the reconstructed comment features for all *n* items, $L^{I}\in R^{n\times k_{2}}$ represents the language vectors collected from the knowledge graph and autoencoder. Considering that the experiments are conducted on MovieLens datasets, the languages of the movies are obtained from open KGs such as DBpedia, and the languages are encoded with the multi-hot method and input into the autoencoder model for learning the hidden representations *L* ^*I*^. The process of *L* ^*I*^ learning is consistent with that of *S* ^*I*^, the details can be seen in the upper right of Figure 2.

Then the $con\left(R^{I},A^{I},S^{I},L^{I}\right)$ is input into the second autoencoder, i.e. a semi-autoencoder, to learn the compressed reconstructed output, the encode stage of the semi-autoencoder can be defined as (7)

$$\xi =f\left(Wcon\left(R^{I},A^{I},S^{I},L^{I}\right)+b\right),$$ (7)

where $W\in R^{\left(m+y+k_{1}+k_{2}\right)\times k}$ and $b_{1}^{I}\in R^{k}$ are the weight matrix and bias item, respectively, *k* is the feature dimension of the hidden layer, and *f* is the sigmoid function for nonlinear activation. Then, the decode stage can be shown as follows:

$$R_{semi}^{\prime }=g\left(W^{\prime }\xi +b^{\prime }\right).$$ (8)

Similarly, where $W^{\prime }\in R^{k\times m}$ and $b_{2}^{I}\in R^{m}$ are the weight matrix and bias item of decoding layer respectively, *g* is the identity function for the activation function. Notably, the SGD (stochastic gradient descent) method is utilized in the semi-autoencoder for model optimization. The details can be seen in the bottom left of Figure 2.

### 4.4 Triple-Autoencoder for Recommendation

From Eqs. 7, 8, we can obviously observe that the output of a semi-autoencoder model is the reconstruction of a certain part of the inputs. When computing the loss function, the result of the semi-autoencoder is a reconstruction of the rating matrix *R* ^*I*^ instead of the whole input $con\left(R^{I},A^{I},S^{I},L^{I}\right)$ , which may result in a performance degradation for recommendation. To this end, we design the third autoencoder model to learn the reconstruction of the whole input, that is triple-autoencoder for the recommendation. The encode and decode stage of the triple-autoencoder can be shown as follows:

$$R^{\prime }=g\left(W_{t}^{\prime }f\left(W_{t}R_{semi}^{\prime }+b_{t}\right)+b_{t}^{\prime }\right).$$ (9)

To avoid over-fitting, the *ℓ*_2_ norm regularization of the weight matrix *W*_*t*_ and $W_{t}^{\prime }$ is added to the objective function, which can be shown as follows:

$$J_{r}=\left({\left\|W_{t}\right\|}_{2}^{2}+{\left\|W_{t}^{\prime }\right\|}_{2}^{2}\right).$$ (10)

Thus, the objective function of the triple-autoencoder can be shown as follows:

$$J_{item}=\;{\left\|\left(R^{\prime }-R_{semi}^{\prime }\right)\right\|}^{2}+\;\alpha J_{r},$$ (11)

where *α* is the trade-off parameter that controls the balance of regularization terms. To minimize the distance between the input $R_{semi}^{\prime }$ and the output *R*′, the deviations are minimized to obtain representations for the recommendation. When the model converges, the output layer of the triple-autoencoder is the prediction matrix *R*′ for the recommendation, the details can be shown in the bottom right of Figure 2. Details of the proposed KGTA are summarized in Algorithm 1.

Algorithm 1 Recommendation with knowledge graph *via* triple-autoencoder (KGTA)

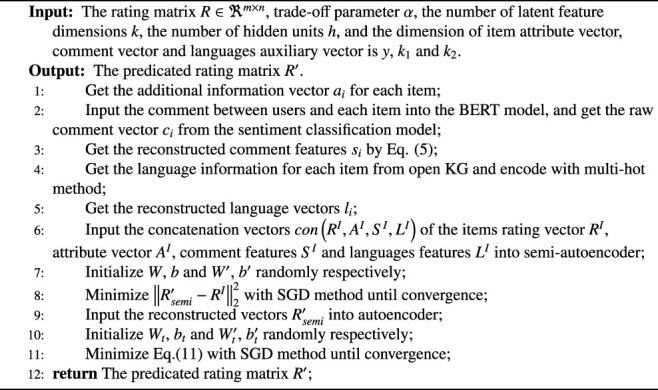

## 5 Experiments

In this section, experiments are conducted on two datasets, MovieLens 100K and MovieLens 1M, to evaluate the effectiveness of our proposed KGTA. In the following, we first introduce the details of two experimental datasets. Secondly, the compared methods, including the MF-based and deep neural network-based methods, are given. In addition, the evaluation metrics such as MAE and RMSE are also presented. Then, the comparative experimental results and their observations are presented in detail. Finally, the main properties such as parameter sensitivity are analyzed for certain datasets.

### 5.1 Datasets

The details of two real-world datasets used in the experiments are listed in Table 2, including rating density, the number of users, items, and ratings.

**TABLE 2:** Details of the three datasets used in our experiments.

Dataset	Number of users	Number of items	Number of ratings	Rating density (%)
MovieLens 100K	943	1,682	100,000	6.3
MovieLens 1M	6,039	3,883	1,000,209	4.27

MovieLens 100K1: it is a well-known and most widely applied dataset for evaluating recommendation performance. There are 943 users and 1,682 movies with 100,000 ratings on a scale of 1–5, and each user rated at least 20 movies. In MovieLens 100K, item attributes such as the title, release date, and genres of movies are also provided for improving recommendation performance.

MovieLens 1M2: It is an enlarged version of the Movielens 100K dataset, which has also been widely applied in the recommendation. It has 6,040 users and 3,706 movies with 1,000,209 ratings. Similar to Movielens 100K, the ratings are scaled from 1 to 5, and auxiliary information such as movie title, release date, and category are also provided.

### 5.2 Compared Methods and Evaluation Metrics

#### 5.2.1 Compared Methods

To evaluate the effectiveness of the proposed KGTA, the following matrix factorization methods, meta-learning methods, and deep neural network methods were conducted:

• Non-negative matrix factorization (NMF) (Lee and Seung, 2001). It is the basic matrix factorization method for the recommendation. In our experiments, we use the generalized Kullback–Leibler divergence as the update rules in NMF.
• Singular value decomposition plus (SVD++) (Koren, 2008). It exploits explicit and implicit feedback from users to combine the latent factor model and the neighborhood model into a unified model for the recommendation.
• Meta-learned user preference estimator (MeLU) (Lee et al., 2019). It estimates user preferences based on a small number of items to alleviate the cold start problem for the recommendation.
• Meta-learning method for cold start recommendation on Heterogeneous Information Networks (MetaHIN) (Lu et al., 2020). It creates a semantic-enhanced task constructor for exploring rich semantics, and a co-adaptation meta-learner with semantic- and task-wise adaptations within each task.
• Neural collaborative filtering (NCF) (He et al., 2017). It is a general recommendation framework that uses designs a deep neural network to learn the interaction between a user and item features.
• Item-based recommendation via autoencoder (AutoRec) (Sedhain et al., 2015). It is the first autoencoder framework in the recommendation, which learns the effective feature representations of items for collaborative filtering.
• Hybrid Collaborative Recommendation via Semi-Autoencoder (HCRSA) (Shuai et al., 2017). It is a hybrid collaborative filtering framework based on the semi-autoencoder, which incorporates auxiliary information for semantic rich representation learning.
• Personalized recommendation with knowledge graph *via* dual-autoencoder (PRKG) (Yang et al., 2021). The side information of items is extracted from DBpedia and encoded into low-dimensional representations in this method, and a semi-autoencoder is introduced to incorporate this auxiliary information for the recommendation.

#### 5.2.2 Implementation Details and Parameter Settings

The PREA toolkit (Lee et al., 2014) is adopted for the implementation of MF-based methods such as NMF and SVD++. For the methods of MeLU, MetaHIN, and HCRSA, we re-compile the source code as 4, 5, and 6. The default parameters of these three methods remain unchanged as reported in the original paper in the MovieLens dataset. For the method AutoRec, we select an item-based autoencoder that can achieve better performance than the user-based autoencoder. For fairness, the parameters of AutoRec and PRKG are consistent with ours in all two datasets. In our experiments, we set *α* = 0.1 after some preliminary tests for all datasets. The maximum number of iterations in gradient descent is set at 300. The number of hidden units is set at 300 for all datasets^5^ ^,^ ^6^.

#### 5.2.3 Evaluation Metrics

In the experiments, we introduced root mean square error (RMSE) to measure the performance of our proposed KGTA and all compared methods in the recommendation, which can be shown as (12). It is worth mentioning that the smaller value of RMSE indicates better results.

$$RMSE=\sqrt{\frac{\underset{r_{u,i}\in TestSet}{\sum }{\left(r_{u,i}-r_{u,i}^{\prime }\right)}^{2}}{\left|TestSet\right|}},$$ (12)

where *r*_*u*,*i*_ and $r_{u,i}^{\prime }$ represent the original rating matrix and the predication matrix, respectively.

### 5.3 Experimental Results

**FIGURE 3:**
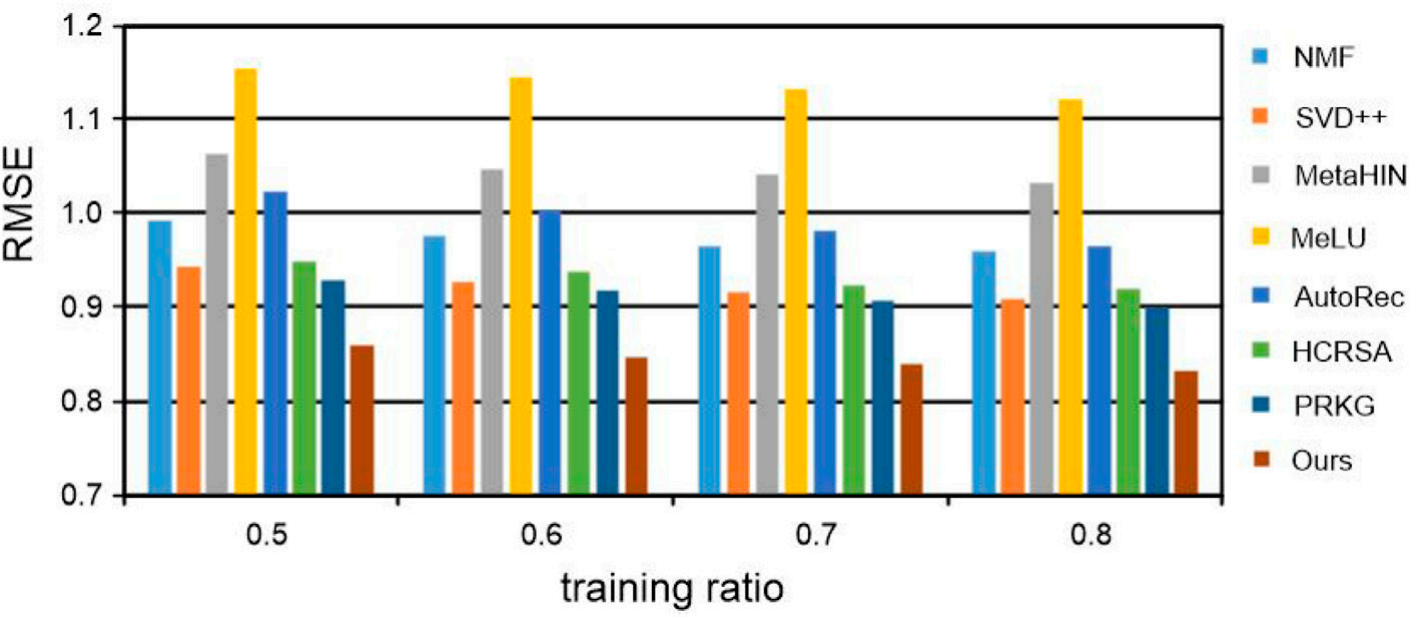
RMSE of our KGTA and compared methods on the MovieLens 100K dataset.

**FIGURE 4:**
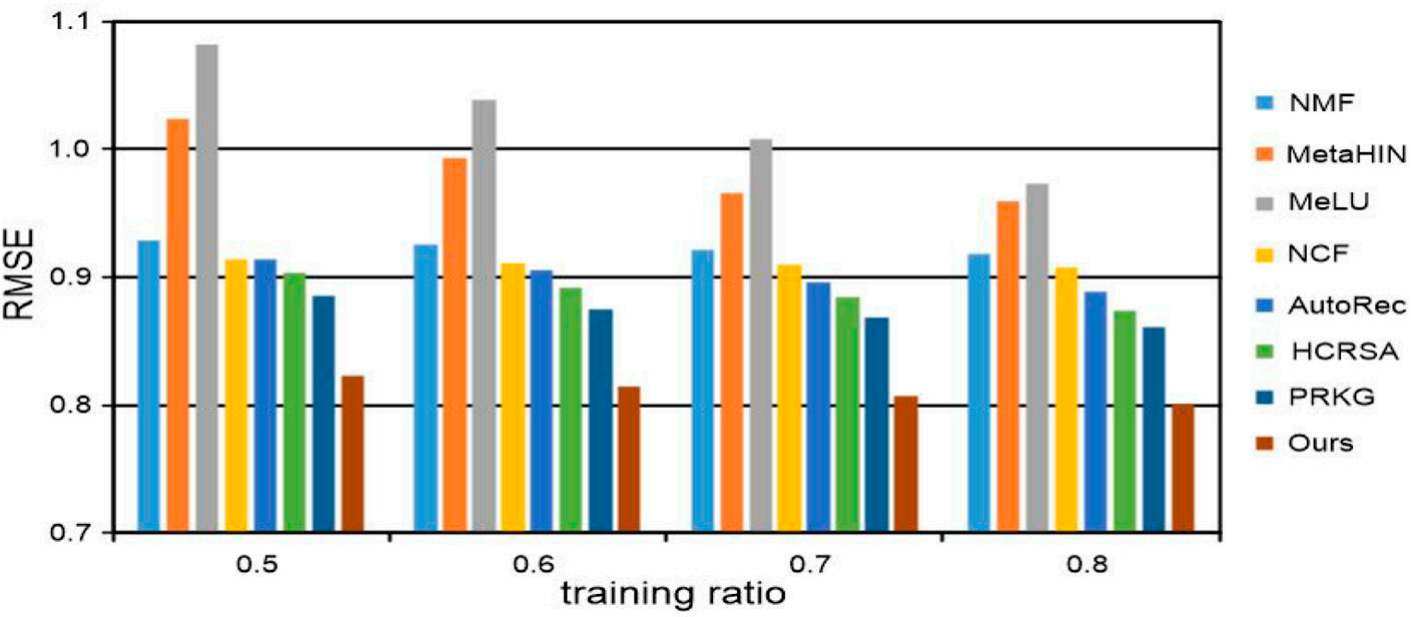
RMSE of our KGTA and compared methods on the MovieLens 1M dataset.

For each data set, the percentages of 50%, 60%, 70%, and 80% are sampled into training data, respectively, and the rest are used for test data. The experimental results of RMSE on the MovieLens 100K and MovieLens 1M datasets are recorded in Table 3 and Figures 3, 4 respectively. Notably, all the results are obtained by repeating the experiments 5 times and taking the average value. From all the results, we have the following insightful observations:

• The performance of all recommended methods is improved with the increase of training data. It is worth mentioning that meta-learning methods such as MetaHIN and MeLU have not changed much, which may be due to the meta-learning methods being designed to alleviate the cold start problem for the recommendation.
• Generally, among the three types of methods, meta-learning methods perform the worst, probably because they are primarily designed to address the cold start problem. The methods for deep neural networks can achieve more desirable performance in most cases than both meta-learning and matrix factorization methods, which reveals the powerful ability of deep neural networks in learning the feature representations for personalized recommendation.
• Among all the deep neural network methods for recommendation, our KGTA is significantly better than NCF and AutoRec, which shows the superiority of introducing auxiliary information for addressing the problem of data sparsity and improving the performance of personalized recommendations.
• In the method of HCRSA, attributes such as the title, release date, and genre of a movie are introduced to the semi-autoencoder model for prediction. From the results listed in Table 3 and Figures 3, 4, we can observe that our KGTA consistently outperforms HCRSA, which demonstrates the superiority of incorporating the key factors of collaborative filtering, such as the comments from users to items, to improve the performance of personalized recommendation.
• Although both the methods introduce auxiliary information, our KGTA outperforms PRKG by up to 7 RMSE points on two well-known datasets, which shows the advantage of designing a serial connection of semi-autoencoder and autoencoder for learning more abstract and higher-level feature representations in the recommendation.
• Overall, the proposed KGTA performs best in all groups, which validates the effectiveness of incorporating the key information between users and items and designing a serial connection of semi-autoencoder and autoencoder for the recommendation. It should be noted that KGTA can achieve stable performance in both MovieLens 100K and MovieLens 1M. These results demonstrate that our KGTA can perform well even if the dataset is sparse.

**TABLE 3:** The performance of RMSE on MovieLens 100K and MovieLens 1M datasets.

Datasets	Methods	Proportion of training data			
MovieLens 100K	-	**50%**	**60%**	**70%**	**80%**
	NMF	0.991	0.976	0.965	0.960
	SVD++	0.943	0.927	0.915	0.909
	MetaHIN	1.062	1.046	1.041	1.032
	MeLU	1.154	1.144	1.132	1.121
	AutoRec	1.023	1.003	0.981	0.964
	HCRSA	0.948	0.937	0.923	0.919
	PRKG	0.928	0.917	0.907	0.899
	KGTA	**0.859**	**0.847**	**0.840**	**0.832**
MovieLens 1M	NMF	0.928	0.925	0.921	0.918
	MetaHIN	1.024	0.993	0.965	0.959
	MeLU	1.082	1.038	1.008	0.973
	NCF	0.914	0.911	0.909	0.907
	AutoRec	0.914	0.905	0.896	0.888
	HCRSA	0.903	0.892	0.884	0.874
	PRKG	0.885	0.875	0.868	0.861
	KGTA	**0.823**	**0.814**	**0.807**	**0.8**

The bold values provided in Table 3 represent the experimental results of our proposed method (KGTA) and are the best results among all the comparison methods.

### 5.4 Parameter Sensitivity

**FIGURE 5:**
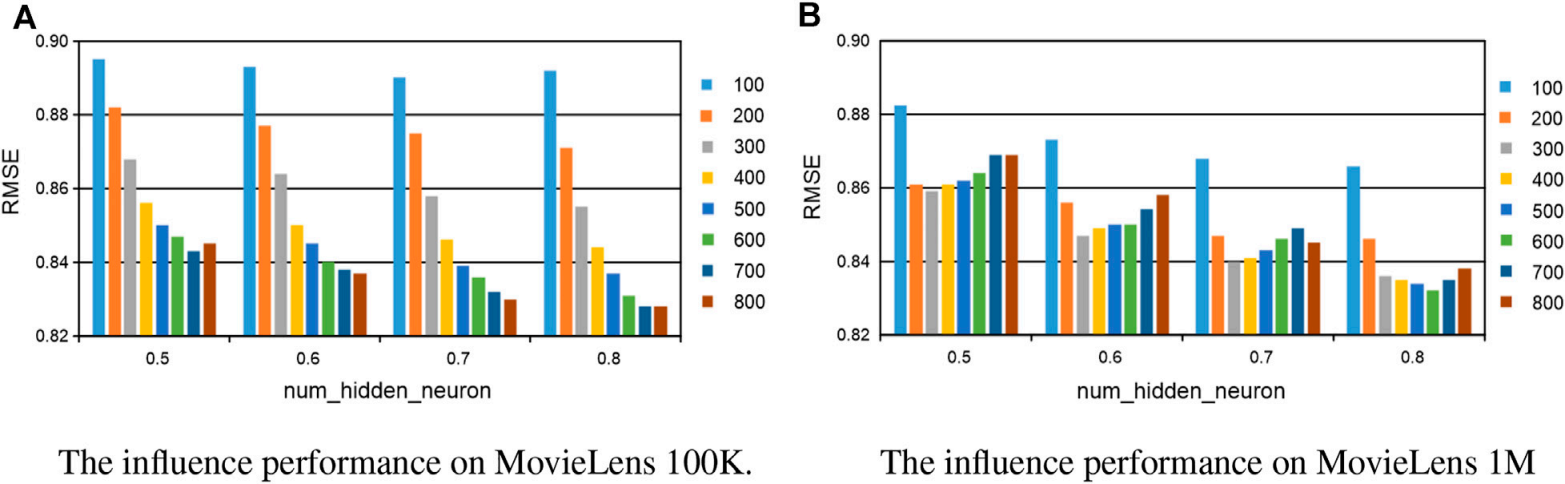
The parameter influence of the number of hidden layer neurons on our KGTA. **(A)** The influence performance on MovieLens 100K. **(B)** The influence performance on MovieLens 1M.

**FIGURE 6:**
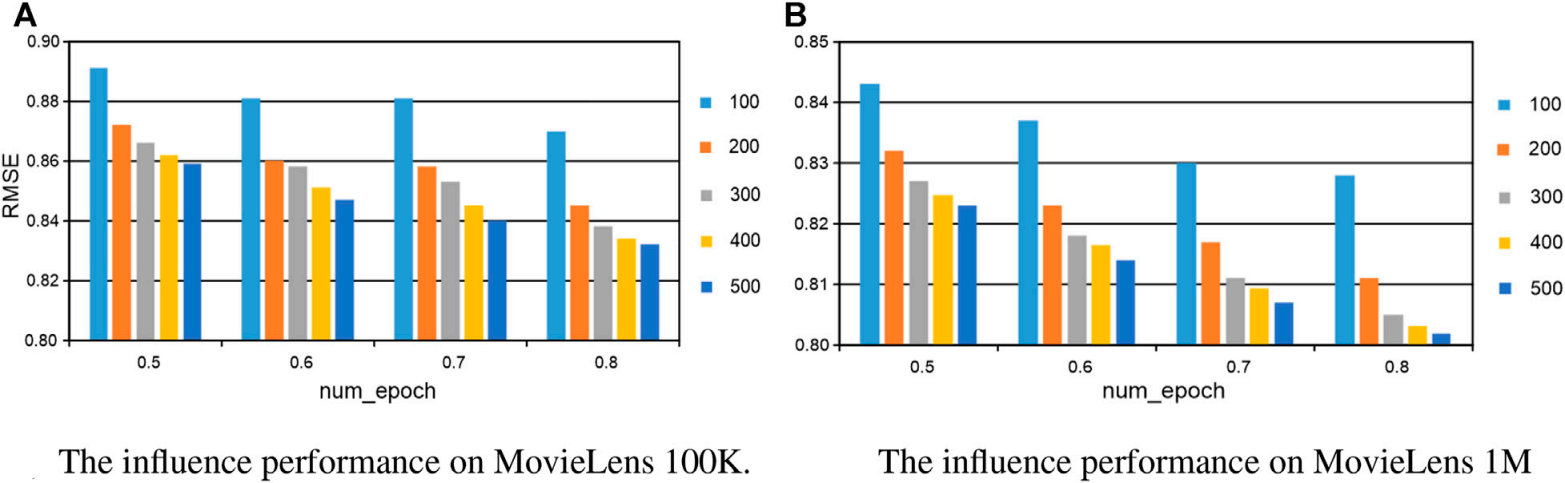
The parameter influence of the number of epochs on our KGTA. **(A)** The influence performance on MovieLens 100K. **(B)** The influence performance on MovieLens 1M.

**FIGURE 7:**
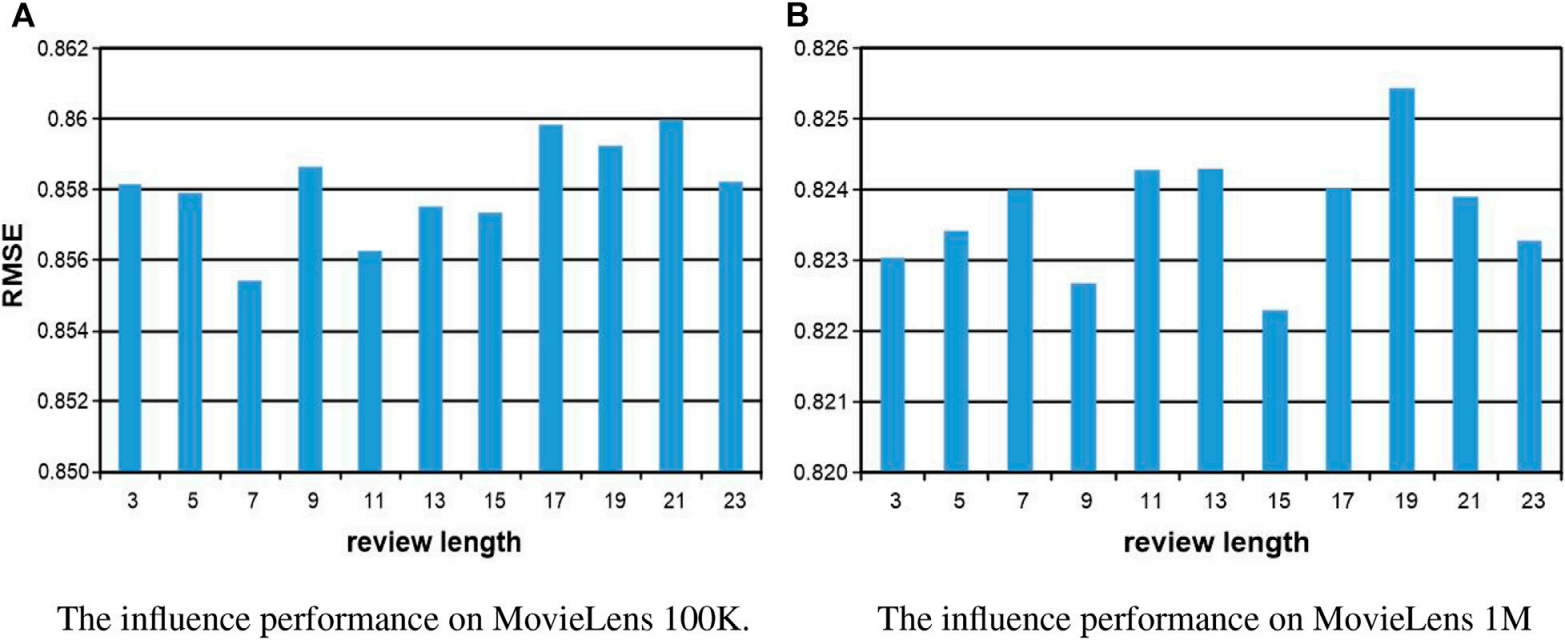
The parameter influence of the length of comments on our KGTA. **(A)** The influence performance on MovieLens 100K. **(B)** The influence performance on MovieLens 1M.

In this section, we investigate the influence of parameters in our proposed method, including the number of hidden layer neurons, the number of epochs, and the length of comments in the training. When one parameter is changed, the others are fixed in the experiments. The number of hidden layer neurons is varied from 100 to 800, the number of epochs is altered from 100 to 500, and the length of comments is sampled from the set {3, 5, 7, 9, 11, 13, 15, 17, 19, 21, and 23}. In the experiments, the validation was conducted on MovieLens 100K and MovieLens 1M, respectively. For the number of hidden layer neurons and the number of epochs, the experiments are conducted with 50%–80% of the training data. All the results are reported in Figures 5, 6, and we set *the number of epoch* = 500 for both datasets, *the number of hidden layer neurons* = 300 and *thenumberofhiddenlayerneurons* = 400 for MovieLens 100K and MovieLens 1M, respectively. For the length of comments, experiments are conducted on 50% of the training data with the best and most stable parameters configuration of the number of hidden layer neurons and epoch, all the results are reported in Figure 7, and we set *the length of comments* = 5 for both the datasets.

## 6 Conclusion

In this paper, we propose a feature representation learning method with a knowledge graph via triple-autoencoder for personalized recommendation called KGTA. We propose a serial connection between the semi-autoencoder and autoencoder methods. In our method, we were able to incorporate side information distilled from DBpedia for more useful item feature representations, and the key factors of collaborative filtering, such as comment information between users and items, are incorporated into the autoencoder as auxiliary information. Moreover, the item-based rating and all the external information are incorporated into the semi-autoencoder to obtain low-dimensional information representation. Finally, the reconstructed output generated by the semi-autoencoder is input into a third autoencoder to learn better feature representations for personalized recommendation. Extensive experiments demonstrate the proposed method outperforms other state-of-the-art methods in effectiveness. In future work, we will try to achieve superior performance by incorporating less information and utilizing an attention network to strengthen the feature integration or without auxiliary information from the open knowledge base.

## Data Availability

The original contributions presented in the study are included in the article/Supplementary Material, further inquiries can be directed to the corresponding authors.

## Footnotes

1
http://files.grouplens.org/datasets/movielens/ml-100k.zip.

2
http://files.grouplens.org/datasets/movielens/ml-1m.zip.

3
http://www.librec.net/download.html.

4
http://github.com/hoyeoplee/MeLU.

5
https://github.com/rootlu/MetaHIN.

6
https://github.com/cheungdaven/semi-ae-recsys.
